# Genome-wide identification and expression analysis of the *WOX* gene family in quinoa (*Chenopodium quinoa* Willd.)

**DOI:** 10.7717/peerj.21040

**Published:** 2026-04-13

**Authors:** Yuanyuan Lan, Shengjiang Wu, Qi Xu, Xufang Jiang, Zhenhua Lu, Xiaofan Huang, Shubo Chang, Bo Ding, Ming Li, Jianchao Hao, Xiaodong Xie, Gaoyi Cao

**Affiliations:** 1 Tianjin Key Laboratory of Intelligent Breeding of Major Crops, College of Agronomy & Resources and Environment, Tianjin Agricultural University, Tianjin, China; 2 Guizhou Academy of Tobacco Science, Upland Flue-Cured Tobacco Quality & Ecology Key Laboratory of China Tobacco, Guiyang, China; 3 Zunyi Tobacco Company of Guizhou Province, Zunyi, China; 4 Hotan Vocational and Technical College, Hetian, Xinjiang, China; 5 Zhangjiachuan Hui Autonomous County Agricultural Technology Extension Station, Tianshui, China

**Keywords:** *Chenopodium quinoa*, *WOX* transcription factors, Gene family, Expression analysis, Stem cell regulation, Regeneration potential, Protein interaction network

## Abstract

*Chenopodium quinoa* exhibits broad-spectrum tolerance to abiotic stresses. However, its limited regenerative capacity hinders systematic functional characterization and the application of gene editing. *WOX* transcription factors play pivotal roles in meristem maintenance, organogenesis and regeneration. Here, we identified 11 *CqWOX* family members from the quinoa genome and conducted phylogenetic, structural, promoter, and expression analyses. The CqWOX proteins showed marked variation in length and physicochemical properties and were predicted to localize to the nucleus. Expression profiling revealed strong tissue- and stage-specific expression, and quantitative reverse transcriptase polymerase chain reaction (qRT-PCR) during callus induction showed non-significant but consistent trends between cotyledonary nodes and hypocotyls. *CqWOX1*, *CqWOX7* and *CqWOX10* showed high expression during seed germination and seedling stages, implying roles in embryonic development and organ differentiation. *CqWOX6* and *CqWOX8* were enriched in meristems, potentially maintaining stem cell zone homeostasis. In 14-day callus, qRT-PCR showed that cotyledonary nodes tended to increase *CqWOX3/4/5/11*, whereas in hypocotyls *CqWOX3* and *CqWOX11* tended to increase and *CqWOX4* and *CqWOX5* to decrease. Abiotic stress assays demonstrated differential responses. *CqWOX8* was significantly upregulated under salt, combined salt-alkali, and low-temperature stresses, suggesting its mediation of broad-spectrum stress signaling integration. *CqWOX3* and *CqWOX10* specifically responded to alkali stress, potentially linking to ion/osmotic homeostasis, whereas *CqWOX2* and *CqWOX9* were suppressed under multiple stresses. Protein interaction predictions indicated that *CqWOX4* may crosstalk with the CLV3/CLE-ARR signaling module to coordinate hormonal and developmental pathways, and collaborate with the BBM-GIF-LEC2 regeneration network to establish a stem cell homeostasis-hormone response-organ regeneration regulatory cascade. Collectively, this study provides a resource for WOX evolution and candidate genes for improving quinoa regeneration and stress resilience.

## Introduction

Quinoa (*Chenopodium quinoa* Willd.), originating from the Andean region of South America, is hailed as a complete nutritional grain due to its comprehensive profile of essential amino acids, abundant unsaturated fatty acids, and soluble dietary fiber. Beyond its nutritional value, quinoa exhibits remarkable tolerance to abiotic stresses such as high salinity, drought, low temperatures, and ultraviolet (UV) radiation (Hinojosa et al., 2018; Abbas et al., 2023). Consequently, it has been designated by the Food and Agriculture Organization as a strategic crop for ensuring food security in the face of climate change(Yadav et al., 2023). In recent years, the yield potential of quinoa on marginal lands, particularly in saline-alkali and arid regions, has been systematically demonstrated, establishing it as a key species within sustainable agricultural systems (Zhou et al., 2024b).

Despite its strong tolerance to abiotic stresses, quinoa production still faces challenges from various biotic and abiotic constraints. For instance, quinoa is highly sensitive to elevated temperatures during the flowering and grain-filling stage. Abnormal high temperatures severely impede the grain-filling process, resulting in poor grain plumpness and consequently significant yield reduction (Abbas et al., 2023). Furthermore, the lack of dedicated herbicides necessitates reliance on manual weeding in commercial production, increasing labor costs. Quinoa plant architecture (*e.g.*, branching pattern, flowering time, plant height) exhibits considerable variation influenced by environmental conditions. Combined with inappropriate cultivation practices or rainy/windy weather, this frequently leads to lodging, hindering mechanized harvesting (Kaur, Sunkaria & Garg, 2022). Moreover, the value of quinoa seedlings and young shoots as a healthy vegetable makes the crop susceptible to pest infestations, such as aphids and thrips. Simultaneously, quinoa shows sensitivity to diseases like root rot, downy mildew, and leaf spot (Hinojosa et al., 2018). These factors collectively hinder the further development of the quinoa industry. To address obstacles in quinoa production and industry development, optimizing overall agronomic performance through modern breeding programs-such as transgenesis and genome editing-offers an effective pathway to generate elite germplasm, breed breakthrough cultivars, and accelerate the industrialization of quinoa.

However, quinoa faces significant technical bottlenecks in genetic transformation and plant regeneration, often being regarded as a recalcitrant transformation species. Major obstacles include low callus induction rates, tissue browning, poor regeneration efficiency, and extended culture cycles. These issues severely hinder the establishment of stable and reproducible transformation systems (Yue et al., 2019; Santhoshi et al., 2025). The lack of an efficient transgenic technology platform restricts the application of gene editing techniques, thereby delaying the breeding process for quinoa varieties with superior agronomic traits. Although virus-induced gene silencing (VIGS) (Zhou et al., 2024b) and protoplast transient expression systems (Li et al., 2022) provide convenient tools for studying gene function in quinoa, these methods are primarily suitable for short-term exploratory research. Due to their unstable expression, transient duration, and tissue specificity, they are insufficient to meet the practical demands of germplasm innovation and long-term trait improvement.

In recent years, the integration of regeneration-associated factors with optimized gene editing systems has significantly enhanced genetic manipulation efficiency in diverse plant species, particularly in traditionally recalcitrant transformation crops. For example, the WUS/BBM co-expression system has substantially increased regeneration frequency in maize and sorghum (Mookkan et al., 2017) and has been incorporated into commercial maize breeding programs. Recent research utilizing a ternary vector system to introduce GRF-GIF fusion proteins has constructed an efficient clustered regularly interspaced short palindromic repeats (CRISPR) gene editing platform, optimizing chromosomal integration efficiency and expression stability of foreign genes in sorghum (Li et al., 2024). In wheat, the GRF-GIF system has effectively enhanced regeneration capacity and shortened culture cycles, facilitating the transition of this crop from “recalcitrant” to an editable model (Debernardi et al., 2020). Furthermore, the application of WUSCHEL-related homeobox (WOX)-type transcription factors (*e.g.*, *TaWOX5*) has been demonstrated to further improve transformation efficiency (Wang et al., 2022a). Concurrently, gene editing technology itself has undergone continuous refinement, enabling successful multiplex gene editing at multiple loci in wheat (Li et al., 2021) and establishing multi-gene editing platforms in crops such as cotton (Sun et al., 2024). These advancements provide viable technological pathways for functional gene research and molecular breeding in quinoa.

WOX is a plant-specific family of homeobox transcription factors whose members share a conserved homeodomain. Within the family, WUS-clade proteins typically contain a WUS-box, and some members also harbor an EAR-like repression motif (LxLxL), which is commonly associated with transcriptional repression. Phylogenetic analyses typically classify *WOX* genes into three clades-ancient, intermediate, and modern (WUS)-providing a framework for functional specialization among family members during development (Van der Graaff, Laux & Rensing, 2009). In model plants, WUS is essential for maintaining stem cells in the shoot apical meristem and forms a negative feedback loop with CLAVATA3 (CLV3) to regulate stem-cell homeostasis (Wu, Li & Kramer, 2019). Functionally, WUS has been experimentally shown to possess both activating and repressive activities as a transcription factor: it acts predominantly as a repressor in stem-cell regulation, but can function as an activator in floral organs (Schoof et al., 2000). During early regeneration, *WOX11/12* are induced near wound sites, promoting partial dedifferentiation of somatic cells and the formation of root founder cells, thereby initiating de novo root organogenesis (Liu et al., 2014; Sheng et al., 2017). Although prior studies have provided preliminary insights into the conservation, distribution patterns, and putative regeneration-related regulatory roles of the quinoa *WOX* gene family, their breadth and depth remain limited as genomic resources continue to improve. Accordingly, we systematically identify *WOX* family members in quinoa and, through an integrative analysis combining phylogenetics, expression profiling, and functional enrichment, investigate the mechanisms by which this family contributes to regeneration, development, and stress responses. Our goal is to provide a theoretical basis for establishing efficient genome-editing and genetic transformation systems in quinoa.

## Materials & Methods

### Identification and sequence analysis of the *WOX* gene family in quinoa

The complete set of *Arabidopsis thaliana WOX* gene family members was retrieved from the TAIR database (https://www.arabidopsis.org, accessed on 3 Dec 2024). The *Chenopodium quinoa* whole-genome data were obtained from the website (https://phytozome-next.jgi.doe.gov/info/Cquinoa_v1_0, accessed on 3 Dec 2024) (Goodstein et al., 2012; Jarvis et al., 2017), and the Swiss-Prot reference database was downloaded from UniProt (https://www.uniprot.org/) (Ahmad et al., 2025). The Hidden Markov Model (HMM) profile for the WOX homeobox domain (PF00046) was acquired from the InterPro database (https://www.ebi.ac.uk/interpro/) (Blum et al., 2025). Putative quinoa *WOX* family members were initially identified through sequence similarity alignment using TBtools II (Chen et al., 2023a), with *Arabidopsis* WOX protein sequences as queries against the quinoa genome. Candidate sequences were subsequently validated by BLASTP alignment against the Swiss-Prot database, followed by manual screening to remove non-specific hits. To ensure comprehensive identification, conserved domain screening was performed by searching the quinoa genome file using the PFAM ID (PF00046) with an *E*-value threshold of <1e−5. The final set of *CqWOX* genes was determined by intersecting the manually curated candidates with the conserved domain-based sequences.For functional annotation, physicochemical properties of the deduced CqWOX proteins were predicted using the ExPASy online toolkit (https://www.expasy.org/) (Duvaud et al., 2021). Subcellular localization was inferred *via* WoLF PSORT (https://wolfpsort.hgc.jp/) (Horton et al., 2007).

### Phylogenetic analysis of the *WOX* gene family in quinoa

WOX candidate protein sequences were retrieved from PlantTFDB for *Physcomitrium patens*, *Amborella trichopoda*, *Oryza sativa*, and *Zea mays*, together with the *Chenopodium quinoa* WOX proteins identified in this study and the *Arabidopsis thaliana* WOX set downloaded from TAIR. Sequences were screened against the Homeobox domain (Pfam: PF00046), retaining only those with a complete homeodomain and removing entries that were evidently incomplete or potentially chimeric. For each gene locus, one representative transcript isoform was kept and renamed according to its transcript index. A maximum-likelihood (ML) phylogenetic tree was inferred in TBtools II, with branch support assessed using 1,000 bootstrap replicates. Tree visualization and annotation were performed with the online tool TVBOT (https://www.chiplot.online/tvbot.html) (Xie et al., 2023).

### Identification of motifs and gene structures in the quinoa *WOX* gene family

Conserved motifs were analyzed using the MEME Suite online tool (https://meme-suite.org/meme/tools/meme) (Bailey et al., 2015) with *Chenopodium quinoa* WOX protein sequences. Conserved domain composition was determined *via* the InterPro database. Gene structure features (exon-intron organization) were extracted and analyzed from the quinoa genome GFF3 annotation file using TBtools II. A phylogenetic tree of the 11 quinoa WOX members was constructed by the Maximum Likelihood (ML) method. Finally, motif, domain, gene structure, and phylogenetic data were integrated for multidimensional correlation analysis through the TBtools II visualization module, enabling evolutionary interpretation of sequence characteristic variations.

### Analysis of *Cis*-acting elements in quinoa *WOX* gene promoters

The 2,000 bp promoter sequences upstream of the transcription start sites for 11 quinoa *WOX* genes were extracted using TBtools II. These sequences were submitted to the PlantCARE database (https://bioinformatics.psb.ugent.be/webtools/plantcare/html/) (Lescot et al., 2002) for *cis*-regulatory element prediction. Results were visualized *via* TBtools II.

### Protein interaction network prediction for quinoa CqWOX4

The protein interaction network of CqWOX4 was predicted using the STRING database (https://string-db.org/) (Szklarczyk et al., 2023). CqWOX4 protein sequences were aligned with the *Arabidopsis thaliana* ortholog WUS to infer conserved interaction partners (Database reference: STRING v12.0).

### Expression profiling of quinoa *WOX* genes and abiotic stress treatments on quinoa seedlings

Seeds of the quinoa cultivar Jin Quinoa 1 were provided by the Tianjin Agricultural University. Uniform, plump seeds were surface-sterilized in 75% (v/v) ethanol for 1 min, rinsed three times with sterile distilled water, and blotted dry on sterilized filter paper. Ten seedlings were sown per pot containing autoclaved substrate and grown in a controlled-environment chamber (16 h light / 8 h dark, 25 ± 2 °C, 60% relative humidity). At the two-leaf stage, seedlings were thinned to four uniform plants per pot. Four-week-old seedlings were randomly assigned to seven treatments: distilled water (control, CK), 150 mM NaCl (low-salt, LS), 400 mM NaCl (high-salt, HS), 150 mM NaHCO_3_ (alkaline stress, A), a 1:1 mixture of NaCl and NaHCO_3_ (saline-alkaline stress, SA), 1 °C chilling (LT), and 37 °C heat stress (HT). Treatment solutions were renewed every 24 h for 5 consecutive days.

### RNA isolation, library preparation and sequencing

Leaf tissues were harvested after treatment, rapidly chilled in liquid nitrogen, and kept at −80 °C until use. Total RNA was isolated using the RNAprep Pure Plant Kit (TransGen, Beijing, China). RNA yield, purity and integrity were evaluated by spectrophotometry (NanoDrop 2000c) and agarose gel electrophoresis. Only RNA passing quality control was carried forward, and one µg RNA per sample was used as the input for subsequent library construction.

### Sample size and power analysis

To determine the number of biological replicates required, we performed a power analysis using the RNASeqPower package (v1.48.0; Bioconductor). Assuming a coefficient of variation of 0.4, a minimum fold change of 2, an average read count of 20 per gene and a desired statistical power of 0.8 at FDR = 0.05, the calculation indicated that at least three biological replicates per group were necessary to detect differentially expressed genes (DEGs). Consequently, we included three independent biological replicates per treatment, and no technical replicates were used because pilot experiments showed that technical variation was negligible (<1%). Sequencing depth was set to ∼6 Gb per sample, which preliminary saturation curves indicated was sufficient to reach >90% gene coverage.

Qualified samples underwent strand-specific RNA-seq (reference-genome strategy) by Biomarker Technologies Corporation. mRNA was purified, cDNA was synthesized, and the sequencing library was prepared following the manufacturers’ recommendations, including adaptor ligation and PCR amplification. Libraries were validated on a Bioanalyzer and sequenced on an Illumina NovaSeq 6000 platform to generate 150 bp paired-end reads.

### RNA-seq data analysis

Low-quality reads were removed using custom Perl scripts, including reads consisting only of adapters, reads with > 5% ambiguous bases (N), and reads in which < 20% of bases were at Q20 (Phred ≥ 20). Clean reads were aligned to the *Chenopodium quinoa* reference genome v1.0 (QQ74/PI 614886) with HISAT2. Gene-level counts were analyzed in DESeq2 (v1.46.0) with median-of-ratios normalization, dispersion estimation, and Wald tests; *P* values were adjusted by the Benjamini–Hochberg procedure. Using the untreated control (CK) as the baseline, log_2_ fold changes (log_2_FC) were computed for each treatment *versus* CK. For visualization only, effect sizes were shrinkage-adjusted, and this step did not affect significance calls. To improve readability of the *WOX* heat map, *WOX* genes with zero counts across all samples were excluded from plotting, while all remaining *WOX* genes were retained. The heat-map matrix comprised log_2_FC values for six stresses (LS, HS, A, SA, LT, HT) relative to CK, with the CK column fixed at 0. Rows were hierarchically clustered on log_2_FC values excluding CK and plotted in R. Cells meeting padj (BH-FDR) ≤ 0.05 and |log_2_FC| ≥ 1 were outlined in black.

Public RNA-seq datasets covering eight quinoa developmental stages/tissues-dry seeds; roots of 2-day-old seedlings; 1-week-old seedlings; shoot apical meristems (SAM) at 2 and 3 weeks; and stems, leaves, and inflorescences of 6-week-old plants-were retrieved from the NCBI Sequence Read Archive. Expression was quantified as TPM, imported into TBtools II, transformed as log_2_(TPM+1), subjected to row-wise Z-score normalization per gene, and hierarchically clustered. The resulting tissue heat map depicts relative, rather than absolute, expression patterns across tissues.

### Replicate information and statistical power

The final dataset comprised three biological replicates per condition (*n* = 3), satisfying the power-analysis requirement (≥3) for detecting ≥2-fold DEGs with 80% power. No technical replicates were included because their inclusion did not improve statistical power beyond the biological replicates.

### Data availability

Raw RNA-seq reads generated in this study have been deposited in the Genome Sequence Archive (GSA) at the National Genomics Data Center (NGDC) under accession CRA028609 (Chen et al., 2021; CNCB-NGDC Members Partners, 2025). Public RNA-seq datasets analyzed in this work were obtained from the NCBI Sequence Read Archive (SRA) under BioProjects PRJNA816061, PRJNA1070813, and PRJNA394651 (Leinonen et al., 2011). To support reproducibility, we provide the processed figure-source table used to plot the stress-response heat map as Table S1.

### Quantitative reverse transcription polymerase chain reaction

Seeds of the quinoa cultivar Jin Quinoa 1 were surface-sterilized by immersion in 75% (v/v) ethanol for 1 min, rinsed once with sterile distilled water, soaked in 2% (w/v) sodium hypochlorite (NaClO) for 15 min, and then rinsed five times with sterile water. Sterilized seeds were sown on half-strength Murashige and Skoog (1/2 MS) medium and grown at 25 °C under a 16 h light/8 h dark photoperiod for 5 days. Aseptic seedlings were dissected to obtain hypocotyls and cotyledonary nodes. These tissues were sampled as the control group. For the treatment (callus-induction) group, dissected hypocotyls and cotyledonary nodes were transferred to callus-induction medium and incubated in darkness at 25 °C for 14 days. All samples were snap-frozen in liquid nitrogen immediately after collection and stored at −80 °C. Three independent biological replicates were collected per group.

Total RNA was isolated using the FastPure Universal Plant Total RNA Isolation Kit (Vazyme, Nanjing, China) according to the manufacturer’s instructions. First-strand cDNA was synthesized with HiScript IV All-in-One Ultra RT SuperMix for qPCR (Vazyme, Nanjing, China). Gene-specific primers for the *Chenopodium quinoa* WOX gene family were designed with Primer3 (Table S2). *CqTUB9* was used as the internal reference gene for normalization. Quantitative reverse transcriptase polymerase chain reaction (qRT-PCR) was performed using a 2 × SYBR Green qPCR Master Mix (Cwbio, Jiangsu, China) on a QuantStudio™ 3 Real-Time PCR System (Applied Biosystems, Waltham, MA, USA). Each 20 µL reaction contained one µL cDNA template, 0.5 µL each of forward and reverse primers (10 µM), 10 µL 2 × SYBR Green Master Mix, and eight µL nuclease-free water. Thermal cycling conditions were: 95 °C for 30 s; followed by 45 cycles of 95 °C for 5 s and 60 °C for 30 s. Each target was assayed with three biological replicates and three technical replicates.Relative transcript abundance was calculated by the 2^∧−ΔΔCt^ method in Microsoft Excel 2021, and results were plotted in the same software. Statistical significance between control and callus-induction groups was assessed using a two-tailed Student’s *t*-test (*P* < 0.05).

## Results

### Genome-wide identification and physicochemical characterization of the quinoa *WOX* gene family

Leveraging the chromosome-scale reference genome of quinoa, we identified 11 *WOX* family members that were sequentially named *CqWOX1*-*CqWOX11* according to their chromosomal order (Table 1). Primary structure analysis revealed that the CqWOX proteins range from 159 to 676 amino acids in length, with CqWOX4 being the largest (676 aa) and CqWOX10 the smallest (159 aa). Correspondingly, the molecular masses varied markedly, peaking at 76.73 kDa for CqWOX4 and bottoming at 18.51 kDa for CqWOX10. Theoretical isoelectric points (pI) spanned 5.27–9.56; CqWOX3 was the most acidic (pI = 5.27), whereas CqWOX6 was the most basic (pI = 9.56), indicating divergent charge properties among paralogs.

**Table 1:** Basic information of *WOX* genes in quinoa and physicochemical properties of its protein.

Gene	Sequence ID	Number of amino acid/aa	Molecular weight/u	Theoretical pI	Instability index	Aliphatic index	Grand average of hydropathicity	Sub-cellular localization
*CqWOX1*	AUR62031114-RA.v1.0	351	37,957.32	6.94	49.87	66.1	−0.433	Nucleus
*CqWOX2*	AUR62012213-RA.v1.0	194	22,247.03	8.5	57.72	55.36	−0.725	Nucleus
*CqWOX3*	AUR62003747-RA.v1.0	286	31,821.42	5.27	62.53	65.8	−0.735	Nucleus
*CqWOX4*	AUR62009597-RA.v1.0	676	76,728.64	6.02	58.21	65.74	−0.761	Nucleus
*CqWOX5*	AUR62001809-RA.v1.0	630	71,230.72	5.53	58.64	66.05	−0.7	Nucleus
*CqWOX6*	AUR62035466-RA.v1.0	233	26,650.89	9.56	53.21	55.67	−1.049	Nucleus
*CqWOX7*	AUR62014909-RA.v1.0	344	37,177.44	6.73	48.12	67.44	−0.439	Nucleus
*CqWOX8*	AUR62031363-RA.v1.0	233	26,734.02	9.47	54.43	57.77	−1.019	Nucleus
*CqWOX9*	AUR62022846-RA.v1.0	178	20,461.97	8.58	59.51	57.08	−0.742	Nucleus
*CqWOX10*	AUR62017610-RA.v1.0	159	18,505.4	8.62	78.69	55.16	−1.232	Nucleus
*CqWOX11*	AUR62002048-RA.v1.0	200	22,682.01	5.6	66.24	55.1	−0.982	Nucleus

Instability index (II) predictions showed that most CqWOX proteins possess II values >40, suggesting poor *in vitro* stability. Aliphatic indices (AI) ranged from 55.10 to 67.44, with CqWOX7 exhibiting the highest AI (67.44), potentially conferring enhanced thermostability. Grand average of hydropathicity (GRAVY) values were negative for all members (−1.232 to −0.439), classifying them as weakly hydrophilic; CqWOX8 was the most hydrophilic (GRAVY = −1.232) and CqWOX7 the least (GRAVY = −0.439), implying functional dependence on the nuclear microenvironment. Subcellular localization predicted that all 11 CqWOX proteins reside exclusively in the nucleus, consistent with their role as transcriptional regulators of plant growth and development.

### Phylogenetic analysis of the *WOX* gene family in quinoa

**Figure 1:**
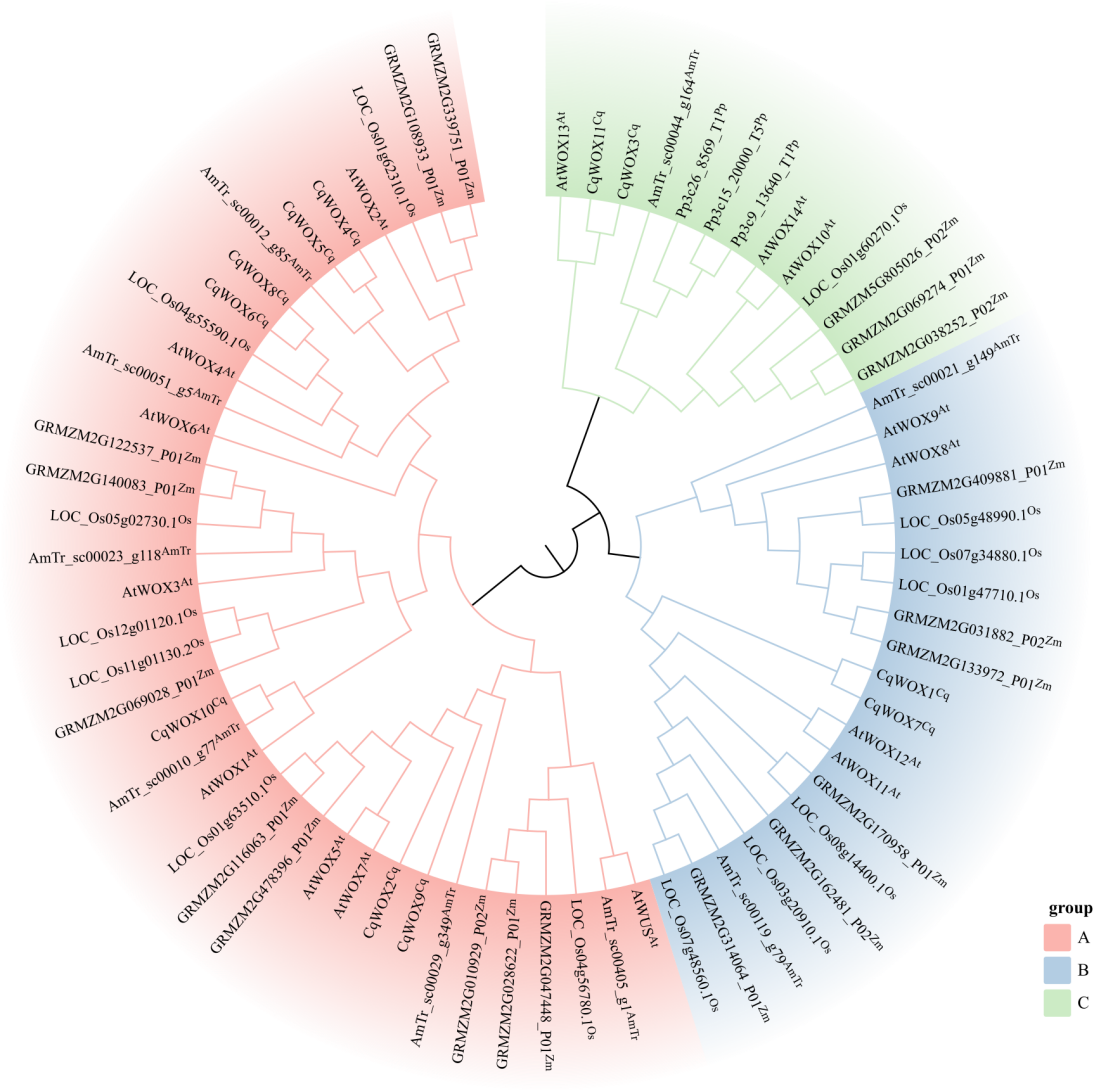
Phylogenetic analysis of WOX proteins. Sequences from *Chenopodium quinoa* (Cq), *Arabidopsis thaliana* (At), *Oryza sativa* (Os), *Zea mays* (Zm), the basal angiosperm *Amborella trichopoda* (AmTr), and the moss *Physcomitrium patens* (Pp) were included. Gene names at the branch tips are suffixed with species abbreviations to indicate their origin. The WOX family is classified into three major clades distinguished by background color: Group A (Red) corresponds to the Modern/WUS clade; Group B (Blue) represents the Intermediate clade; and Group C (Green) corresponds to the Ancient clade.

Following the canonical three-clade framework of the *WOX* family (Van der Graaff, Laux & Rensing, 2009) and large-scale phylogenetic evidence (Wu, Li & Kramer, 2019), the protein phylogeny was partitioned according to the *Arabidopsis*-based classification into the modern, intermediate, and ancient clades. The tree includes *Chenopodium quinoa*, *Arabidopsis thaliana*, *Oryza sativa*, *Zea mays*, the basal angiosperm *Amborella trichopoda*, and the moss *Physcomitrium patens*. Within this framework, the placements of quinoa genes are as follows (Fig. 1): A (modern/WUS), B (intermediate), and C (ancient).In the modern clade (Group A), CqWOX6 and CqWOX8 form a sister pair adjacent to *O. sativa* LOC_Os04g55590.1 and AtWOX4; CqWOX4 and CqWOX5 form a sister pair and cluster with AtWOX2 and grass orthologs from *O. sativa* and *Z. mays*; CqWOX2 and CqWOX9 fall within the broader WUS/WOX5/WOX7 assemblage but are not the immediate sister to WUS; CqWOX10 is close to AtWOX1 together with the homolog from *A. trichopoda*. In the intermediate clade (Group B), CqWOX1 and CqWOX7 constitute a stable pair embedded within the WOX11/12-like group and match AtWOX11/12 and cereal counterparts. In the ancient clade (Group C), CqWOX11 and CqWOX3 lie near AtWOX13, whereas *P. patens* occupies deeper, long-branched positions and *A. trichopoda* frequently marks outlying nodes, aiding in defining the clade depth. Overall correspondence with the *Arabidopsis* groupings indicates that the quinoa WOX family retains a conserved subdivision within the angiosperm framework, while the modern clade shows species-specific expansion characterized by paired paralogs.

### Gene architecture and conserved motif profiling of the *CqWOX* transcription factor family

**Figure 2:**
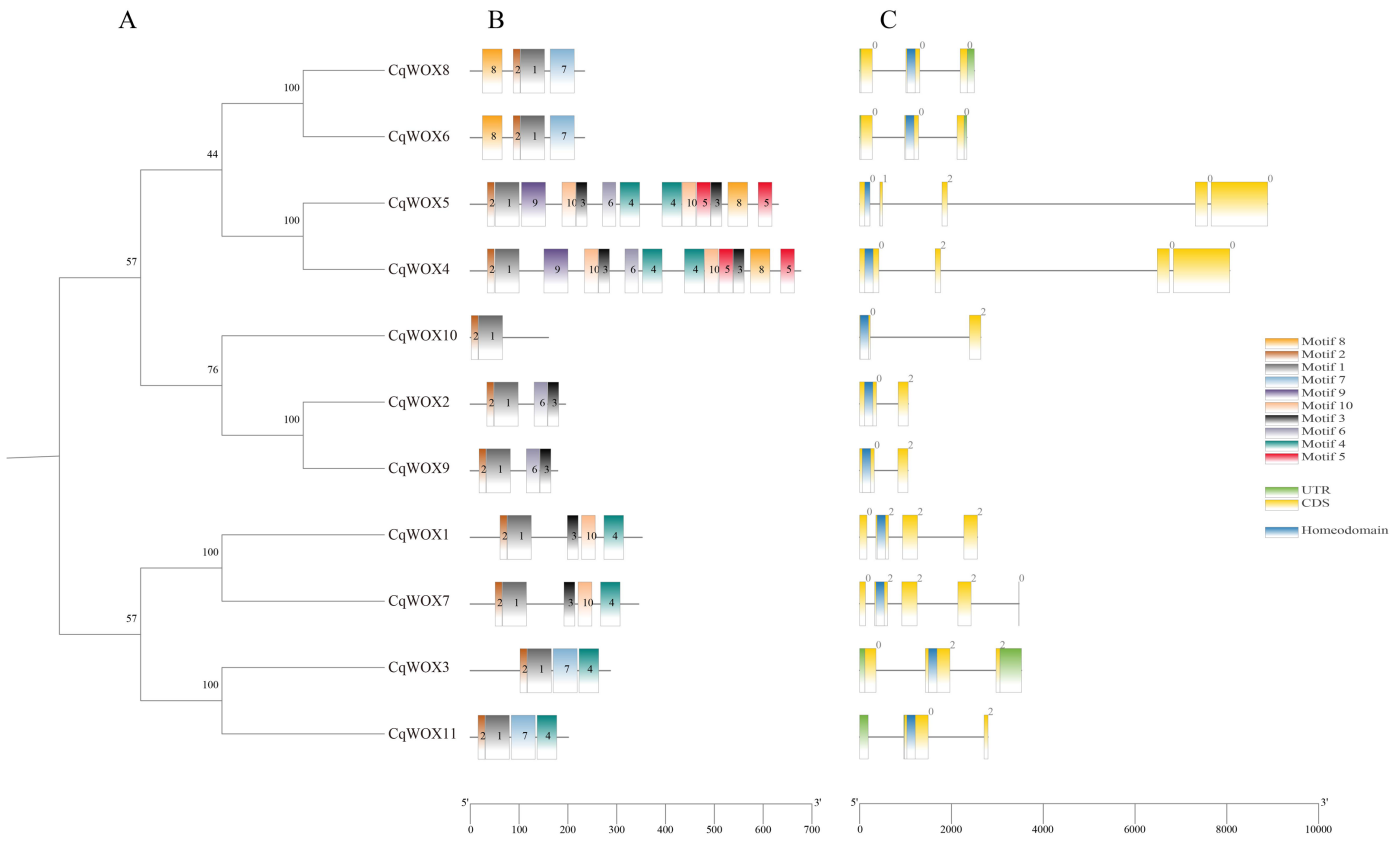
Evolutionary relationships, exon-intron architecture, conserved motifs, and domains of the 11 CqWOXs. (A) Phylogenetic tree of CqWOXs; (B) distribution and positions of conserved motifs within CqWOX proteins; (C) exon–intron organization and conserved domains.

To systematically dissect the conserved architecture and functional potential of the quinoa WUSCHEL-related homeobox (*WOX*) gene family, the coding sequences (CDS) and protein motifs of eleven *CqWOX* members were comprehensively analyzed (Fig. 2). The phylogenetic tree resolved the *CqWOX* genes into three sub-clades, with *CqWOX1*/*7*/*3*/*11* clustering together, implying close evolutionary ties and possibly convergent functions. Domain scanning revealed that all CqWOX proteins harbor a highly conserved homeodomain that mediates DNA binding and constitutes the core functional module of the *WOX* family. MEME-based motif discovery identified ten signature motifs (motifs 1-10); motifs 1 and 2 are universally present and form the functional backbone, whereas CqWOX4 and CqWOX5 additionally possess motifs 4, 5, 6 and 9, suggesting involvement in more intricate regulatory networks. At the gene-structure level, exon-intron organizations diverge markedly: *CqWOX1* and CqWOX3 exhibit short introns and compact CDS regions, whereas *CqWOX4* and *CqWOX5* contain long introns and multiple exons, likely reflecting sophisticated transcriptional control. *CqWOX11* further carries 5′ or 3′ UTRs (green), indicating potential post-transcriptional regulatory capacity.

### *Cis*-regulatory element profiling of the *CqWOX* promoters

**Figure 3:**
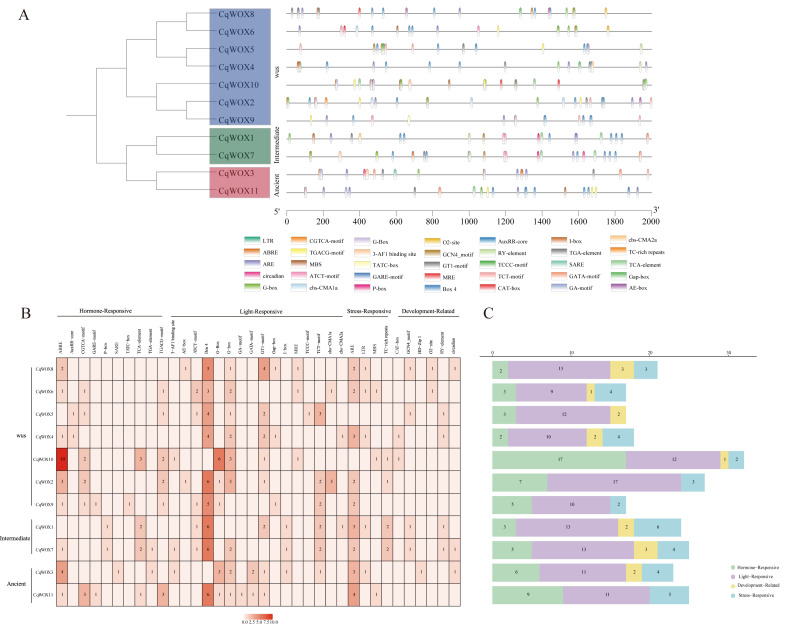
*Cis*-acting elements in *CqWOX* transcription factor promoters. (A) Distribution of*cis*-acting elements in *CqWOXs* promoters. (B) Abundance of*cis*-acting elements per *CqWOX* member (darker colors indicate higher element counts).(C) Stacked classification of*cis*-acting element types: hormone-responsive (green), photoresponsive (purple), growth/development-related (yellow), and stress-responsive (blue) elements.

To clarify the potential roles of the quinoa *CqWOX* family in development and abiotic stress, we extracted the 2-kb regions upstream of the ATG for all 11 *CqWOX* genes and scanned cis-regulatory elements using PlantCARE. As shown in Fig. 3, element distributions are uneven across promoters: light-responsive elements are most abundant, followed by hormone-responsive and then stress-responsive elements; development/organ-related elements account for the remainder. *CqWOX10* contains the highest total number of elements (32), whereas *CqWOX6* and *CqWOX9* each contain 17. All promoters carry light-responsive motifs, with Box 4, G-box, and GT1-motif occurring most frequently, indicating broad regulation by light. Except for *CqWOX5*, all members include stress-responsive motifs such as MBS, LTR, and TC-rich repeats, pointing to links with drought, low-temperature, and defense pathways. For hormone-related motifs, ABRE is most common (25 sites), followed by MeJA-related motifs (CGTCA/TGACG; 20), salicylic acid (TCA-element; eight), gibberellin (GARE/P-box/TATC-box; five), and auxin (AuxRR-core/TGA-element; four). At the gene level, hormone-responsive elements are most numerous in *CqWOX10* (17), followed by *CqWOX11* (nine), *CqWOX2* (seven), and CqWOX3 (six) (Figs. 3B–3C), suggesting participation of these genes in hormone-regulated processes during quinoa development. Development/organ-related motifs also show gene-specific patterns: RY-elements occur in *CqWOX1/4/5/7,* CAT-box in *CqWOX10/4,* O2-site in *CqWOX6/8*, and GATA-motif in *CqWOX11/3* (Fig. 3B). Together, these results indicate that *CqWOX* genes are involved in quinoa growth and are associated with responses to abiotic stress.

### Tissue-specific expression and abiotic stress responses of the *CqWOX* gene family

**Figure 4:**
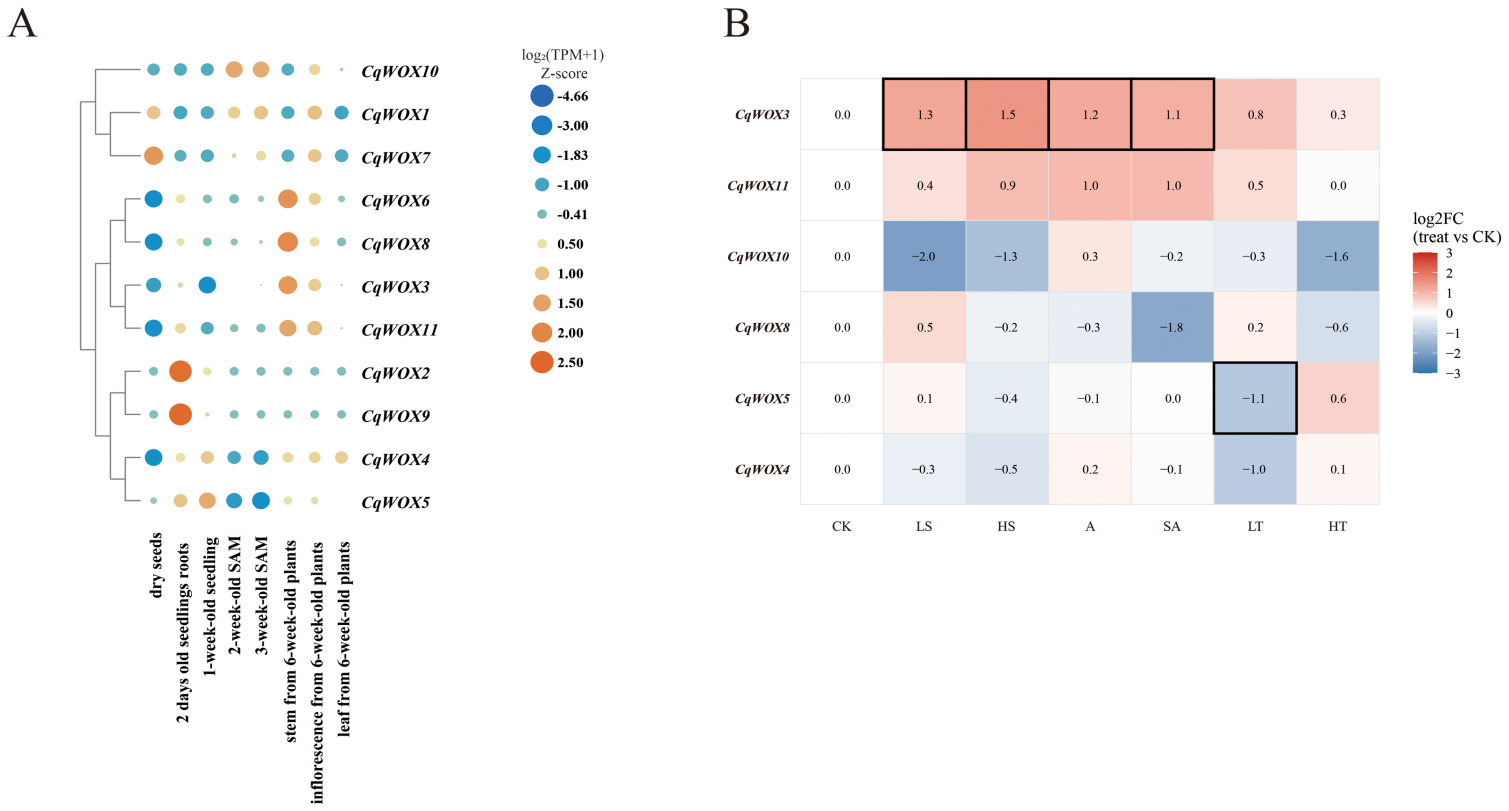
Heat map of the expression profiles of the *CqWOX* gene family across different developmental stages and under abiotic stresses in quinoa. (A) Developmental stages: dry seed, root from 2-day-germinated seed, 1-week germinated seed, 2-week shoot apical meristem (SAM), 3-week SAM, 6-week leaf, 6-week inflorescence, and 6-week stem. Color intensity (orange → blue) denotes relative expression (high →low), and circle size indicates the within-row (*i.e.,* within-gene) relative magnitude. Values are plotted from log_2_ (TPM+1) after row-wise z-score normalization; colors indicate relative levels within each row rather than absolute expression, with rows clustered. (B) Abiotic stresses: control (CK), 150 mM NaCl (LS), 400 mM NaCl (HS), 150 mM NaHCO_3_ (A), NaCl:NaHCO_3_ = 1:1 (SA), 1 °C low temperature (LT), and 37 °C high temperature (HT). Colors shift toward blue for lower expression and toward red for higher expression. Significance was called using dual thresholds of FDR ≤ 0.05 and |log_2_ FC|≥ 1 (≥ 2-fold); cells meeting both criteria are outlined in black.

To compare expression differences of the *CqWOX* family across developmental stages and tissues, we compiled quinoa transcriptomes from NCBI covering eight stages/tissues and generated a relative heat map shown in Fig. 4A. *CqWOX5*, *CqWOX3* and *CqWOX7* are relatively higher in dry seeds; *CqWOX6*, *CqWOX3*, *CqWOX11* and *CqWOX8* are higher in 2-day roots, whereas *CqWOX2* and *CqWOX9* are low; *CqWOX5* and *CqWOX4* are higher in 1-week seedlings; *CqWOX10* is highest in 2–3-week SAM with *CqWOX3*, *CqWOX6*, *CqWOX8* and *CqWOX11* at intermediate levels; in 6-week stems, *CqWOX6* and *CqWOX8* are the highest, followed by *CqWOX3* and *CqWOX11*; in inflorescences, *CqWOX1*, *CqWOX3* and *CqWOX11* are higher; in leaves, *CqWOX3*, *CqWOX11*, *CqWOX4* and *CqWOX5* are intermediate, while *CqWOX1* and *CqWOX7* are near zero. *CqWOX2* and *CqWOX9* remain low across most stages/tissues, with only low-level expression in early roots and 1-week seedlings. Overall, *CqWOX* genes show clear stage/tissue preferences: some members (*e.g.*, *CqWOX10*, *CqWOX6/8)* are enriched in specific meristems or organs, whereas *CqWOX2/9* are generally low.

In addition to tissue/stage differences, we also examined expression responses under stress conditions (Fig. 4B). Using 4-week seedlings treated for 5 days under LS, HS, A, SA, LT and HT relative to CK, the data show that *CqWOX3* is significantly up-regulated under LS, HS, A and SA (log_2_FC 1.1−1.5; FDR≤0.03) and only modestly increased under LT and HT; *CqWOX5* is significantly down-regulated under LT (log_2_FC≈−1.1; FDR≈0.025). *CqWOX11* shows moderate increases under HS, A and SA (log_2_FC 0.9−1.0; FDR <0.001) and a small increase under LT (log_2_FC ∼0.54; FDR ∼0.021), but these do not reach the two-fold threshold; *CqWOX10* tends to decrease under LS, HS and HT (minimum ∼−2.0), *CqWOX8* decreases most under SA (∼−1.8), and *CqWOX4* shows slight decreases across several treatments; these trends do not simultaneously satisfy the |log2FC| and FDR criteria. Taken together with Fig. 4A, the *CqWOX* family displays both stage/tissue preferences and treatment-specific responses: *CqWOX3* is consistently induced by salt/alkaline conditions, *CqWOX5* is repressed by cold, and other members show trends that do not meet the significance criteria.

### Quantitative reverse transcription PCR

**Figure 5:**
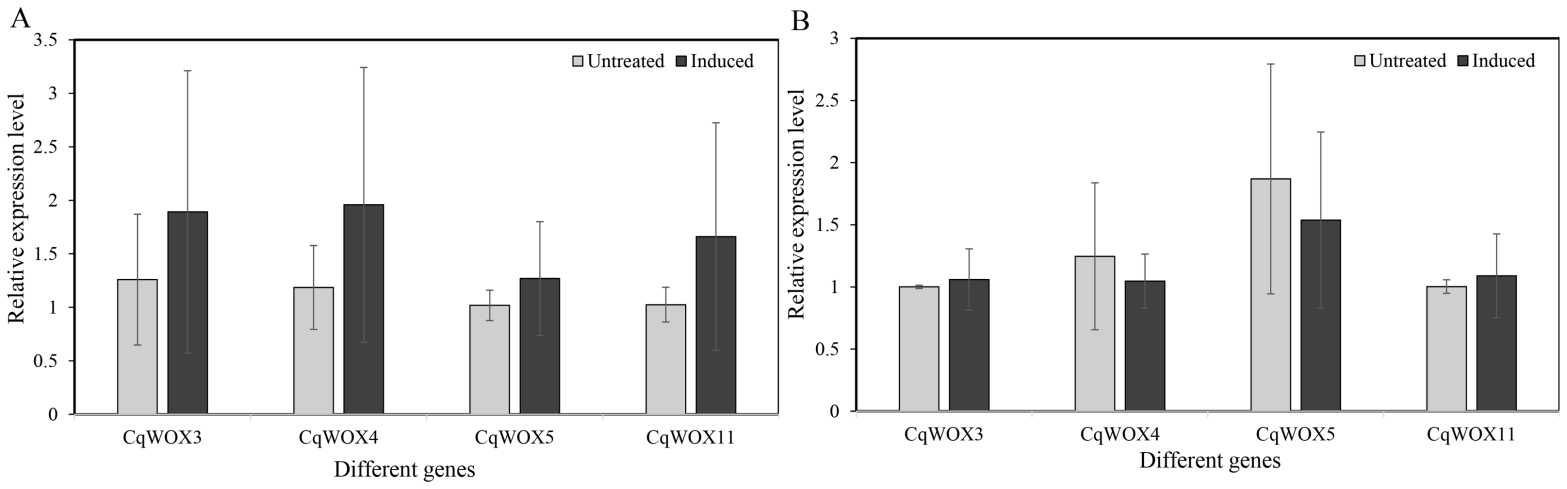
QRT-PCR analysis of four CqWOX genes under callus-inducing conditions. (A) Cotyledonary nodes; (B) Hypocotyls. Bars show relative expression (2^−ΔΔCt^); values are mean ± SE (*n* = 3 biological replicates; technical triplicates were averaged within samples). Light gray, non-induced; dark gray, callus induction for 14 days.

To further examine the association between *WOX* genes and regeneration in quinoa, callus was induced from the cotyledonary nodes and hypocotyls of 5-day aseptic seedlings. After 14 days of induction, qRT-PCR revealed changes in the expression of *CqWOX3*, *CqWOX4*, *CqWOX5*, and *CqWOX11*. In cotyledonary nodes (Fig. 5A), all four genes showed a general trend toward up-regulation relative to the corresponding controls. In hypocotyls (Fig. 5B), *CqWOX3* and *CqWOX11* tended to increase, whereas *CqWOX4* and *CqWOX5* tended to decrease, compared with controls. None of these differences reached statistical significance. Overall, these non-significant trends were broadly concordant with the RNA-seq patterns and may reflect early remodeling of WOX expression during dedifferentiation; however, they should be interpreted as observational and require validation with larger sample sizes.

### Functional protein–protein interaction network of CqWOX

**Figure 6:**
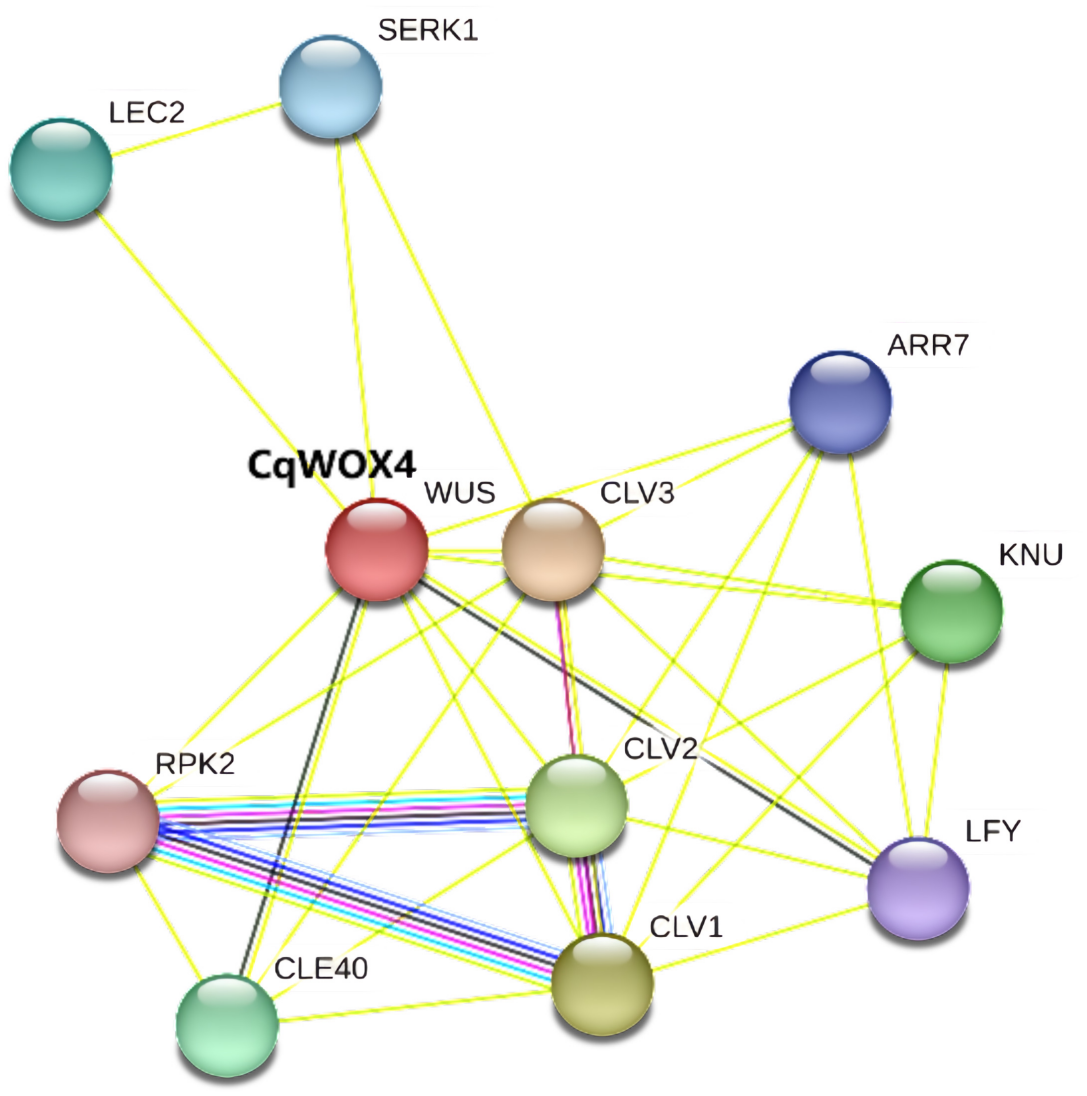
Functional interaction network of CqWOX4 predicted based on Arabidopsis homologs. Functional protein–protein interaction network of CqWOX4 predicted in STRING based on the Arabidopsis WUS ortholog, showing extensive connections to CLV1/2/3, CLE peptides (*e.g.*, CLE40) and their receptor kinases RPK2 and SERK1, the cytokinin-response regulator ARR7, and floral regulators LFY and KNU, thereby supporting a role for CqWOX4 as a central hub linking CLV–WUS and CLE–RPK/SERK signaling with hormone and floral developmental pathways to coordinate stem-cell homeostasis, meristem maintenance, and organogenesis.

To explore potential interaction partners of CqWOX4, we used *Arabidopsis thaliana* as a reference model and constructed an orthology-based protein-protein interaction (PPI) network using the STRING database (Fig. 6). The analysis predicted associations between CqWOX4 and proteins implicated in meristem maintenance and development regulation, including CLAVATA pathway components (CLV1, CLV2, CLV3), CLAVATA3 (CLV3)/Embryo Surrounding Region (CLE) peptides (*e.g.*, CLE40) with receptor kinases (RPK2, SERK1), and regulators linked to cytokinin and floral development (ARR7, LFY, KNU) (Wang, Wu & Sun, 2024). Because these links are inferred from cross-species orthology and database evidence, they represent testable hypotheses rather than experimentally verified interactions.

## Discussion

### Phylogeny, structural architecture and functional evolution of the quinoa *WOX* gene family

Leveraging the chromosome-scale quinoa genome, we identified eleven bona fide WOX members, all harboring a canonical homeobox domain and predicted nuclear localization, consistent with their identity as transcription factors. Phylogenetic reconstruction resolved the CqWOX repertoire into three principal clades-modern, ancient and intermediate-mirroring the branching patterns observed in *Arabidopsis*, rice, apple and other model or crop species, thereby underscoring the deep evolutionary conservation of this family across land plants. Gene-structure and motif analyses revealed that every CqWOX protein possesses the core Motifs 1 and 2, yet exhibits pronounced variation in exon-intron architecture and the complement of ancillary motifs. The core homeodomain is broadly conserved, whereas accessory motifs and gene structures differ; this indicates that while the basic transcriptional function is retained, some CqWOX paralogs have diverged in their modes of regulation. Previous studies have established that Motif 1 corresponds to the homeodomain that mediates DNA binding and target specificity, whereas auxiliary, variable motifs interact with transcriptional co-activators or repressors to confer tissue-specific or stress-responsive modulation (Song et al., 2025). Thus, the invariant Motifs 1/2 secure the fundamental nuclear transcriptional role of CqWOX proteins, whereas structural and motif diversity furnish the molecular basis for functional specialization in organogenesis, developmental progression and environmental adaptation.

### Potential regulatory roles of *CqWOX* genes in abiotic stress responses

A systematic analysis of promoter *cis*-elements shows that the 2-kb regions upstream of the ATG in *CqWOX* genes exhibit uneven distributions across categories: light-responsive elements are most abundant, followed by hormone-responsive, then stress-responsive, with development/organ-related elements being least frequent. As shown in Fig. 3, these motifs cover core pathways related to ABA, MeJA, low temperature, drought, and salinity (Dong et al., 2023). For hormone-related motifs, ABRE is the most common (25 sites), followed by MeJA-related motifs (CGTCA/TGACG; 20), salicylic acid TCA-element (eight), gibberellin-related (GARE/P-box/TATC-box; five), and auxin-related (AuxRR-core/TGA-element; four). At the gene level, *CqWOX10* contains the most hormone-responsive motifs (17), followed by *CqWOX11* (nine), *CqWOX2* (seven), and CqWOX3 (six). In total motif counts, *CqWOX10* is highest (32), whereas *CqWOX6* and *CqWOX9* each contain 17. In addition, stress-responsive motifs (MBS, LTR, TC-rich repeats) are present in all members except *CqWOX5*, indicating connections to drought, low-temperature, and defense pathways. This distribution pattern is consistent with reports in tea and rapeseed, where WOX promoters are also rich in ABA and MeJA responsive elements and are significantly up-regulated under drought, salinity, and low-temperature stresses (Wang et al., 2018; Wang et al., 2019). Furthermore, studies in *Arabidopsis*, rice, and tomato indicate that *WOX* genes integrate hormone signaling and can drive transcriptional reprogramming under diverse stress stimuli (Zhou et al., 2017; Wang et al., 2021a; Chen et al., 2023a; Chen et al., 2023b). Collectively, the motif landscape of *CqWOX* promoters provides molecular clues to their roles in stress adaptation and a framework for dissecting upstream regulators.

Notably, the presence of *cis*-elements indicates regulatory potential but does not guarantee transcriptional activation. Stress- or hormone-responsive expression depends on additional regulatory layers (*e.g.*, chromatin accessibility/epigenetic state, tissue- and stage-specific availability of transcription factors, and combinatorial binding), which may explain discrepancies between motif predictions and observed expression responses.

### Spatiotemporal specificity of *CqWOX* expression and its multifunctional roles in quinoa development, regeneration, and stress adaptation

*CqWOX* genes display clear spatiotemporal partitioning in quinoa. Systematic profiling shows that *CqWOX5/3/7* are relatively higher in dry seeds and *CqWOX5/4* are higher in 1-week seedlings, whereas *CqWOX10* peaks in the 2–3-week shoot apical meristem (SAM) rather than in bulk seedlings; *CqWOX2/9* remain generally low. *CqWOX6/8* are enriched in 2-day roots, and *CqWOX6/8* reach high levels in 6-week stems; in the SAM, *CqWOX6/8* (together with *CqWOX3/11*) are at intermediate levels, with *CqWOX10* highest. These patterns suggest potential roles in embryogenesis and organ primordia formation and are consistent with the conserved WOX-mediated regulation of apical meristem fate in model plants (Breuninger et al., 2008; Lopes, Galvan-Ampudia & Landrein, 2021; Zhang et al., 2025a; Zhang et al., 2025b).

In regeneration-related tissues, *CqWOX* distribution might be associated with regenerative potential. After fourteen days of callus induction, cotyledonary nodes show an overall increase for *CqWOX3*, *CqWOX4*, *CqWOX5*, and *CqWOX11*. In hypocotyls, *CqWOX3* and *CqWOX11* increase, whereas *CqWOX4* and *CqWOX5* decrease. These comparisons do not reach statistical significance, but the directions match the transcriptome. Species use different transformation routes that align with these spatial expression patterns and with genotype and hormone sensitivity. Rice often uses mature embryos. Maize and wheat favor embryogenic callus from immature embryos. Tobacco commonly relies on leaf-disc regeneration (Lopes, Galvan-Ampudia & Landrein, 2021; Zhang et al., 2025a).

WOX, BBM, and the GRF-GIF module are widely recognized as key regulators of somatic embryogenesis and reprogramming. Ectopic BBM is sufficient to trigger somatic embryo formation, and GRF-GIF enhances regeneration by promoting cell expansion and mitosis (Kerstens et al., 2024; Debernardi et al., 2020; Lowe et al., 2016). Methodologically, transient and stable systems are typically combined. VIGS and virus-mediated overexpression allow rapid, reversible gene modulation in recalcitrant materials, while stable transformation supports long-term phenotyping and network reconstruction (Kobayashi et al., 2025; Sidorov, Maughan & Yang, 2024). For breeding, precise activation of WOX or BBM during critical regenerative windows through tissue-specific or chemically inducible promoters has helped overcome transformation bottlenecks in species such as sorghum and wheat (Butler et al., 2025; Wang et al., 2022). Advances in single-cell transcriptomics and spatiotemporal atlases will further define dynamic expression features and regulatory networks across distinct regeneration trajectories (Liu et al., 2024).

For abiotic stress, salt treatments used two NaCl levels. We set 150 mM as LS and 400 mM as HS. Alkaline stress used 150 mM NaHCO_3_. Combined saline-alkaline stress mixed NaCl and NaHCO_3_ at a one-to-one ratio. Low temperature was set to 1 °C and high temperature to 37 °C. The family shows type-specific and dose-dependent responses. *CqWOX3* is significantly up-regulated under LS, HS, A, and SA, with log_2_FC of 1.1 to 1.5 and FDR not exceeding 0.03, and shows only small increases under low and high temperature. CqWOX5 is significantly down-regulated under low temperature, with log_2_FC around −1.1 and FDR around 0.025. *CqWOX11* increases moderately under HS, A, and SA, and increases slightly under low temperature; the magnitude does not reach a two-fold change and FDR is below 0.001. *CqWOX10* decreases under LS, HS, and high temperature, reaching about −2.0 at minimum. *CqWOX8* shows the strongest decrease under combined saline-alkaline stress at about −1.8. *CqWOX4* shows mild decreases under several treatments, and several trends do not meet both the magnitude and FDR thresholds simultaneously. Cross-species studies report that multiple rice *OsWOX* genes respond to salt and low temperature, poplar *PagWOX11/12a* positively regulates salt tolerance, and barley *HvWOX8* promotes germination and root elongation under salt stress; under high temperature, WUS is down-regulated in the *Arabidopsis* shoot apex to enhance thermotolerance (Cheng et al., 2014; Zhang et al., 2025a; Zhang et al., 2025b; Liu et al., 2024). These stress responses overlap in time with expression in meristems and seeds, suggesting shared regulatory programs for organogenesis and environmental adaptation (Gong et al., 2024).

Overall, the *CqWOX* family participates in developmental programming and abiotic-stress responses through differentiated expression, providing clear entry points for functional validation and mechanistic study.

### *CqWOX4*-mediated stem cell fate regulation and regenerative network coordination

Protein interaction network predictions suggest that *CqWOX4* may be connected to CLV3/CLE signaling modules and ARR transcription factors, raising the possibility that it participates in meristematic stem cell regulation and organogenesis. This aligns with the conserved role of *WOX* genes in stem cell homeostasis maintenance (Dolzblasz et al., 2016; Zhou et al., 2024a; Yu et al., 2022). The potential *CqWOX4*-*ARR7* interaction suggests cytokinin signaling mediation for meristem activity regulation, consistent with recent evidence on hormonal control of regeneration. Further analysis reveals possible *WOX*-*BBM*-*GIF* co-regulation: *BBM* induces somatic embryogenesis, while *GIF1* cooperates with *GRF*/*WOX* to promote cell proliferation and stem cell maintenance. *CqWOX4*’s interactome with key developmental factors (*SERK1*, *LEC2*, *KNU*, *LFY*) in quinoa implies multifunctional integration-extending beyond shoot apical meristem maintenance to floral organ formation and regenerative pathway coordination. Thus, *CqWOX4* likely constructs a meristem homeostasis-hormone response-regeneration initiation tripartite network, with its synergy with *BBM*/*GIF* providing a molecular basis for establishing efficient genetic transformation systems. Future gene editing and expression modulation studies are needed to decipher its regulatory logic.

In summary, the quinoa *WOX* family exhibits high structural functional conservation alongside member-specific divergence, potentially linked to environmental adaptation and developmental demands. However, it must be noted that the present study relies primarily on genome-wide identification, transcriptomic profiling, and predictive network analysis, which provide correlative rather than direct functional evidence. While the expression patterns and orthology-based predictions suggest potential roles in regeneration and stress adaptation, functional verification of *CqWOX* genes will require future approaches such as VIGS or CRISPR-mediated gene editing. Nonetheless, this study advances the understanding of *WOX* evolution and potential functions, providing a theoretical basis and identifying target genes for the genetic enhancement of quinoa regeneration capacity and stress resilience.

## Conclusions

Using the quinoa reference genome v1.0, we identified 11 *CqWOX* genes and carried out a systematic analysis of their sequences, phylogeny, gene structures, conserved motifs, promoters, and expression. The phylogeny follows the accepted three-clade scheme (modern, intermediate, ancient) and also points to species-specific expansion in the modern clade, seen as paired paralogs. Structurally, all members share a highly conserved homeobox, while exon–intron layouts and accessory motifs vary, most notably in *CqWOX4/5.* All proteins are predicted to localize to the nucleus, consistent with their roles as transcription factors.

At the promoter level, hormone-responsive elements are broadly enriched, with ABRE most frequent and MeJA-related motifs next, alongside light-responsive sites and stress-related elements for drought, salt, and low temperature. *CqWOX10* carries the highest total number of sites and the most hormone-related sites, suggesting stronger hormone regulation. Expression profiles show clear spatiotemporal specificity: *CqWOX10* is enriched in the shoot apical meristem, *CqWOX6/8* are higher in stems, and *CqWOX2/9* are generally low. Under abiotic stress, *CqWOX3* is significantly induced by saline and alkaline treatments and also increases under high-salt conditions, whereas *CqWOX5* is significantly repressed by cold. Other members mainly show trends that do not pass the combined |log2FC| and FDR thresholds, underscoring gene- and treatment-specific regulation. Consistent with the RNA-seq, 14-day callus qRT-PCR (nodes/hypocotyls from 5-day seedlings) showed non-significant trends: all four genes increased in nodes; in hypocotyls, *CqWOX3/11* tended to rise and *CqWOX4/5* to decline. These patterns may reflect early remodeling of the WOX network during dedifferentiation.

Orthology-based interaction predictions place CqWOX4 at the intersection of the CLV–WUS and CLE–RPK/SERK modules and link it to ARR7, LFY, and KNU, suggesting a regulatory hub that integrates meristem stem-cell maintenance with hormone and floral signaling.

## Supplemental Information

10.7717/peerj.21040/supp-1 Supplemental Information 1 Supporting data underlying the expression heatmap Gene expression data underlying the construction of the tissue expression heatmap.

10.7717/peerj.21040/supp-2 Supplemental Information 2 Primers used for qPCR qPCR primers for CqTUB9, CqWOX3, CqWOX4, CqWOX5, and CqWOX11.

10.7717/peerj.21040/supp-3 Supplemental Information 3 MIQE Checklist

## Additional Information and Declarations

### DNA Deposition

The following information was supplied regarding the deposition of DNA sequences: CqWOX genes was obtained based on quinoa genome annotation information *via* Cq_PI614886_gene_V1_pseudomolecule.gff: https://phytozome-next.jgi.doe.gov/info/Cquinoa_v1_0. The RNA sequencing data of different tissues of quinoa under different treatments are available at NCBI: PRJNA816061, PRJNA1070813 and PRJNA394651.

### Data Availability

The following information was supplied regarding data availability: The raw sequence is available in the Genome Sequence Archive (Genomics, Proteomics & Bioinformatics 2021) in National Genomics Data Center (Nucleic Acids Res 2022), China National Center for Bioinformation/Beijing Institute of Genomics, Chinese Academy of Sciences: CRA028609.
